# A Genome-Wide Association Study of the Chest Circumference Trait in Xinjiang Donkeys Based on Whole-Genome Sequencing Technology

**DOI:** 10.3390/genes14051081

**Published:** 2023-05-14

**Authors:** Ling-Ling Liu, Bin Chen, Sheng-Lei Chen, Wu-Jun Liu

**Affiliations:** Department of Animal Science, Xinjiang Agricultural University, Urumqi 830052, China; linglingliu1988@xjau.edu.cn (L.-L.L.); chenbin_1998@163.com (B.C.); goldliving@outlook.com (S.-L.C.)

**Keywords:** chest circumference, Xinjiang donkey, GWAS, PLINK, key genes

## Abstract

Animal genotyping by means of genome-wide association studies is important for connecting phenotypes of interest with their underlying genetics in livestock. However, the use of whole genome sequencing to investigate chest circumference (CC) in donkeys has rarely been reported. We aimed to use the genome-wide association study approach to detect significant single nucleotide polymorphisms (SNPs) and key genes associated with chest circumference traits in Xinjiang donkeys. We assessed 112 Xinjiang donkeys in this study. The chest circumference of each was measured 2 h before milking. We re-sequenced blood samples from the Xinjiang donkeys, and genome-wide association study analyses were performed using a mixed model with the PLINK, GEMMA, and REGENIE programs. We tested 38 donkeys for candidate SNPs for genome-wide association study using three software programs. Additionally, 18 SNP markers reached genome-wide significance (*p* < 1.61 × 10^−9^). On the basis of these, 41 genes were identified. Previously proposed candidate genes for CC traits were supported by this study, including *NFATC2* (Nuclear Factor of Activated T Cells 2), *PROP1* (PROP Paired-Like Homeobox 1), *UBB* (Ubiquitin B), and *HAND2* (Heart and Neural Crest Derivatives Expressed 2). These promising candidates provide a valuable resource for validating potential meat production genes and will facilitate the development of high-yielding Xinjiang donkey breeds through marker-assisted selection or gene editing.

## 1. Introduction

The donkey (*Equus asinus*), a descendant of the African wild ass, is a common domestic animal [1]. Donkey domestication has been essential to human culture and development, and has played an important role in economic and social life throughout human history [2]. Donkeys provide meat and milk, especially donkey-hide gelatin, which mainly consists of collagen, and is a traditional medicinal material [3]. Donkey meat is widely believed to be palatable because of its high linoleic acid content [4]. Donkeys are principally used as pack animals or for draught work in transport and agriculture. However, the population of donkeys is drastically declining, owing to the mechanization of agriculture and the development of different means of transportation [5,6,7]. The donkey has been used as a working animal for at least 5000 years. There are approximately 50.5 million donkeys globally [8]. The latest China Statistical Yearbook showed that the total stock of donkeys in China was 1.967 million in 2022, about 13.6% of which were distributed in Xinjiang province (data.stats.gov.cn). The Xinjiang donkey is an indigenous breed. Its body is short, with a slightly larger head, erect ears, broad forehead, short nose, and hair in the ear shell; its coat color is mostly gray or black, and there are slight differences in the external characteristics of Xinjiang donkeys in different regions. However, they all have a mild temperament, strong disease resistance, and strong environmental adaptability [9].

Previous studies (from the last two years) have focused on milk from the Halari donkey [10], donkey semen cryopreservation [11], the characterization of donkey-meat flavor profiles [12], the number of thoracolumbar vertebrae and carcass traits in the Dezhou donkey [13], disease [14], the hoof morphometry of donkeys in Pakistan [15], circadian rhythm [16], tendon healing [17], and skin thickness in Dezhou donkeys [18]. However, there have been few studies on the body size traits of donkeys. Body size traits are very important characteristics in animals, and influence sports performance. The main differences in breeding goals can be observed with respect to the functional posterior triangle, as well as regarding size and shape. Breeding plans of different species implement morphological measures as selection goals because of their correlation with production traits. Growth traits, including chest circumference, are important quantitative traits, and have a vital impact on profitability. Hence, improving the growth traits of donkeys, including chest circumference, has become a focus of research in the field of animal husbandry.

Some studies on chest circumference traits have focused mainly on humans, pigs, birds, and horses, but few have addressed donkeys. Wang et al. found that the *NCAPG-LCORL* on chromosome 3 may be a candidate region for the small body size trait in Liangzhou donkeys [19]. Wang et al. used sequencing technology, and found that the *LCORL* gene was associated with chest circumference in Dezhou donkeys [20]. Some studies have found high correlations between infant CC traits and body weight [21,22,23]. Akaboshi et al. [24] found that CC was associated with obesity in young children. Therefore, CC may be a useful marker for rapid growth and help clinicians identify children with obesity. Xu et al. [25] conducted a GWAS using a multi-trait meta-analysis and a linear mixed model based on whole-genome sequencing, and characterized the *PLAG1* gene as being relevant to CC traits. Marelli et al. [26] investigated the variability within turkey CC traits in two Italian heritage breeds. Inoue et al. [27] found that CC traits were significantly correlated with age in Noma horses. The association analysis of donkey CC traits (*p* = 0.013) with *TBX3* polymorphisms showed significant differences between the AA and GG genotypes in the g.3624A > G single nucleotide polymorphism (SNP) locus [28]. Lai et al. [29] found that IGF1-1 loci were significantly associated with the CC of male Dezhou donkeys. Additionally, Wang et al. [30] used polyacrylamide gel electrophoresis to genotype 380 Dezhou donkeys. They identified *CDKL5* as a candidate gene for CC. Recently, it has been shown that improving important economic traits in livestock may benefit from a better understanding of the genetic architecture underlying phenotypes of interest. From this perspective, animal genotyping using GWAS has been recognized as a powerful approach for reconnecting the phenotype of interest with the underlying genetics in livestock species [31].

In the last decade, various GWAS studies have been undertaken on livestock, including socially affected traits and body mass index in Yorkshire pigs [32,33], coat color traits in Arab camels and cattle [34,35], meat quality in camels [36], foot and leg conformation traits in Chinese Holstein cattle [37], milk fatty acid traits in Comisana sheep [38], litter size in sheep [39], body weight traits in yaks [40], growth traits in Braunvieh cattle [41], and growth traits in broilers [42]. Whole-genome sequencing has been used to detect population structures and to identify polymorphisms that might affect the economic traits of livestock animals [43]. However, owing to the lack of a complete chromosome-level reference genome, most recent studies on donkeys are based on mitochondrial levels. The entire genome variation of donkeys has been largely unexplored [44]. Renaud et al. [45] used Chicago HiRise assembly technology to produce a high-quality genome assembly for donkeys. The application of genome-wide association analysis of CC in donkeys has rarely been reported. A single genome-wide association study has been reported, which was performed to identify the genomic variations associated with body size in Yangyuan donkeys [46].

Therefore, this study aimed to detect significant SNPs associated with CC traits in Xinjiang donkeys using the GWAS approach. Using the SNP positions, we also aimed to identify candidate genes and pathways that may influence this trait. Our study provides molecular markers for the selective breeding of CC traits in Xinjiang donkeys.

## 2. Materials and Methods

### 2.1. Experimental Animals and DNA Resequencing Data

**Figure 1:**
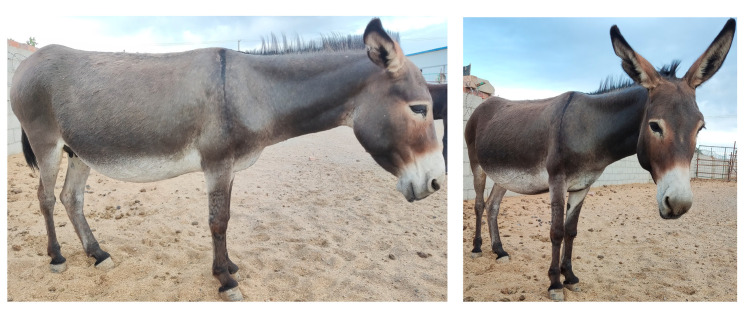
Xinjiang donkey.

In this study, 112 Xinjiang donkeys were collected from Yuepuhu County, Kashgar Region, China. The characteristics of the Xinjiang donkey are shown in Figure 1. Whole blood samples (5 mL) were collected from each Xinjiang donkey using an EDTA anticoagulated blood collection tube and stored at −80 °C. The donkeys in this study were all females, and were 5–7 years old. The pedigree information of all donkeys was unknown. The donkeys were from the same farm, and were kept under standard conditions, with the same diet and management conditions. DNA was extracted using an Animal Blood/Cell/Tissue Genomic DNA Extraction Kit (Tiangen Biochemical Technology Company, Beijing, China). The fragments of sheared DNA were end-repaired, A-tailed, adaptor-ligated, and amplified using Agilent 2100 bioanalyzer (Agilent Technologies, Santa Clara, CA, USA). Paired-end sequencing was performed using the NovaSeq 6000 system (Illumina Inc., San Diego, CA, USA). For accurate quantification of DNA concentration, three methods were used to detect DNA: (1) agarose gel electrophoresis for DNA purity and integrity; (2) nanodrop detection of DNA purity (OD 260/280 ratio); and (3) Qubit 2.0.

We performed a per-base-sequence quality check using Fastp (version 0.19.7) software. The paired-end sequence reads were mapped against the reference donkey genome [*Equus asinus* (ass)] using BWA (version 0.7.17) [47]. Using the “REMOVE_DUPLICATES =true” option in the “mark duplicates” command-line tool of Picard (http://broadinstitu-te.github.io/picard, accessed on 10 February 2022), the potential PCR duplicates were filtered. Samtools (version 1.9) was used for mapping [48]. SNP variant loci were detected using GATK (version 3.8) [49]. Data processing involved discarding of paired reads if: (1) either read contained adapter contamination; (2) more than 10% of bases were uncertain in either read; (3) the proportion of low-quality (Phred quality < 5) bases was greater than 50% in either read.

### 2.2. Phenotype Data Collection

The CC data of 112 Xinjiang donkeys were used for subsequent association analyses. Measurements were taken by the same person using a tape measure and measuring stick, always on the right side of the animal, with the donkey standing with its front and hind legs perpendicular to the ground. Chest circumference measurements were performed after milking.

### 2.3. Variant Site Filtering

SNP filtering was conducted using PLINK software [50] with the following criteria: (1) SNP call rate > 80%; (2) Hardy–Weinberg equilibrium *p*-value < 0.01; and (3) minor allele frequency (MAF) > 0.05.

### 2.4. Genome-Wide Association Study

Single-SNP association analysis was performed using a mixed-model approach in three software packages: PLINK, REGENIE [51], and GEMMA [52]. The year was fitted as a covariate. The following statistical model was used.

y=Wα+Xsβs+g+e

where y is an n × 1 vector of phenotype values for all individuals (CC); W is an n × c matrix of covariates (fixed effects containing the birth year, parity, and mean); α is a c × 1 vector of the corresponding coefficients, including the intercept; Xs is an n × 1 vector of genotypes of a marker at the locus tested; βs is the effect size of the marker; g is an n × 1 vector of random polygenic effects with a covariance structure as g~N(0, $\sigma_{A}^{2}\varphi$); and e is an n × 1 vector of residual errors with e~N (0, $\sigma_{e}^{2}I$), where I is an n × n identity matrix and $\sigma_{e}^{2}I$ is the residual variance. The thresholds of the Bonferroni-corrected *p*-values for suggestive genome-wide significant associations were set at 0.05/6,205,008 (i.e., 8.06 × 10^−9^), and a genome-wide Bonferroni correction threshold of 0.01/6,205,008 (i.e., 1.61 × 10^−9^) was used to assess the significance level for each SNP.

### 2.5. Gene Function Annotation

The variants were annotated using snpEff (version 4.0e) [53] according to the annotation of the National Center for Biotechnology Information (NCBI). The NR database was used to annotate the genes. Genecards (https://www.genecards.org, accessed on 10 February 2022) was used to find the gene function. We selected 200 kb before and after the locus as the final association interval and used the database for functional gene mining of the association interval.

## 3. Results

### 3.1. Descriptive Statistics

The descriptive statistics of the CC trait are reported in Table 1.

It can be seen from Table 1 that the average chest circumference of the 112 Xinjiang donkeys was 151.5 cm, with a maximum value of 180 cm and a minimum value of 130 cm. The chest circumference of Dezhou donkeys is 143–145 cm [20], and that of Hetian green donkeys is 135 cm. The average chest circumference of Yangyuan donkeys is 138.62 cm [46]. The coefficient of variation is an index showing the density rate in relation to the average. Meanwhile, the coefficient of variation of the chest circumference trait is an index for determining the minimum sample size. In our study, the coefficient of variation obtained for chest circumference demonstrated low variability, at 8.44.

**Figure 2:**
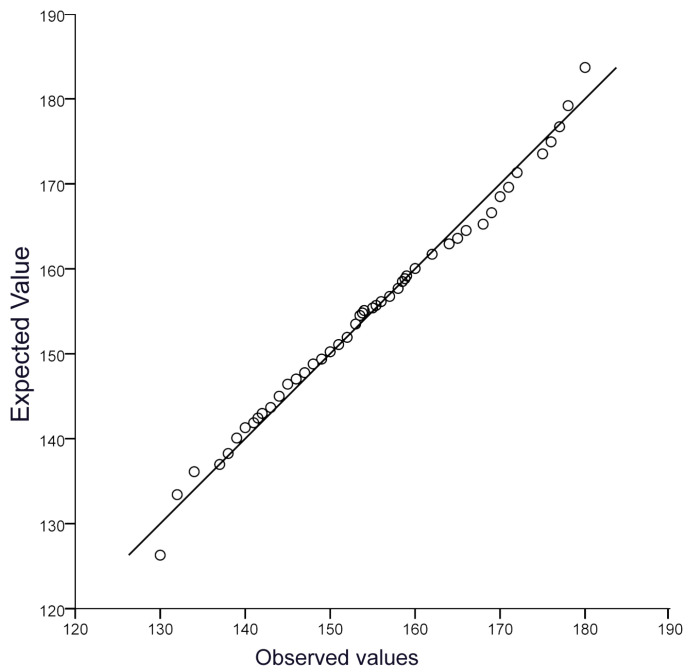
The QQ plot of the CC trait.

We examined the normality of the chest circumference values in Xinjiang donkeys (Figure 2) and found that it conformed to a normal distribution, making it possible to perform further genome-wide association analysis.

### 3.2. Resequencing of Xinjiang Donkeys

We generated the genomic sequences of 112 Xinjiang donkeys, which yielded 2986.2 Gb of clean data with an average depth of 9.3× for subsequent analysis (Table S1). An average of 96.26% (95.05–96.85%) of the proper reads were mapped to the reference genome (Table S2).

After SNP calling and quality control, 12,039,909 high-quality SNPs were obtained for all 112 individuals. Following SnpEff annotation, we found that the number of SNPs located in the intergenic region was the greatest (5,201,198, 43.20%), followed by those in the intron (4,738,320, 39.36%), upstream gene regions (898,713, 7.46%), and downstream gene regions (730,993, 6.07%) (Table S3).

### 3.3. Statistics of Sequencing Coverage Depth

**Figure 3:**
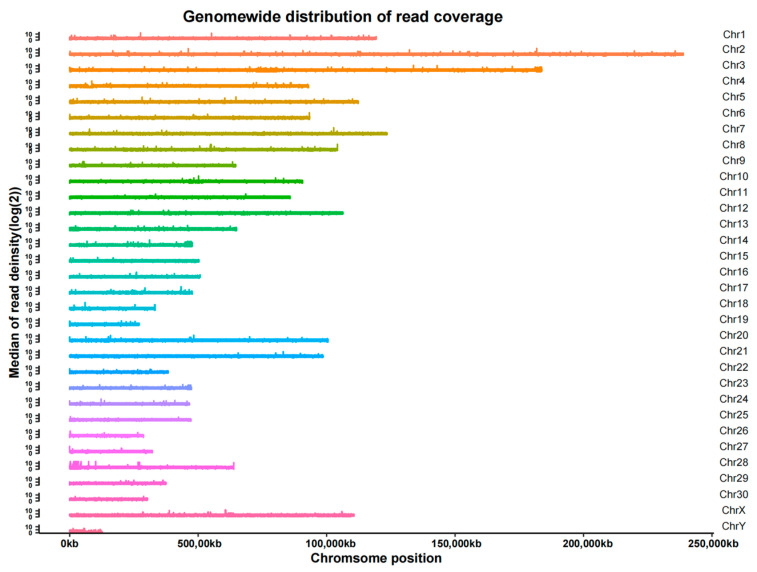
Distribution of read coverage.

After localizing the reads to the reference genome, it was found that the coverage reached an average of 98.49% for 1×, 88.09% for 5× and 47.50% for 10× (Table S4). In addition, we found that the sequencing data were evenly distributed throughout the genome, indicating good sequencing randomness (Figure 3).

### 3.4. QQ Plot Analysis

**Figure 4:**
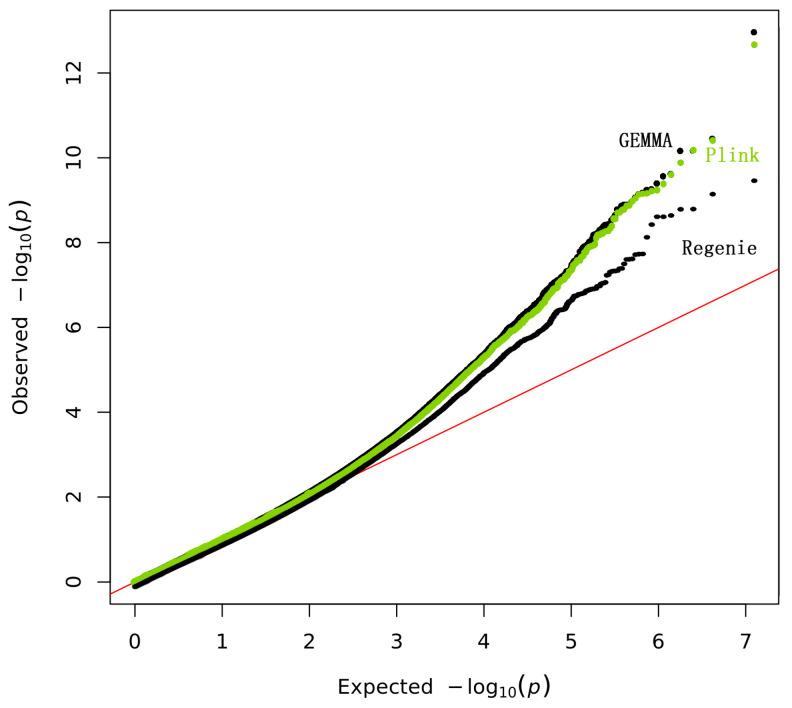
The QQ plots for the GWAS analysis.

The QQ plot of GWAS *p*-values for PLINK, REGENIE, and GEMMA clearly showed the REGENIE correction for the population structure within this population (Figure 4).

### 3.5. Association Analysis

**Figure 5:**
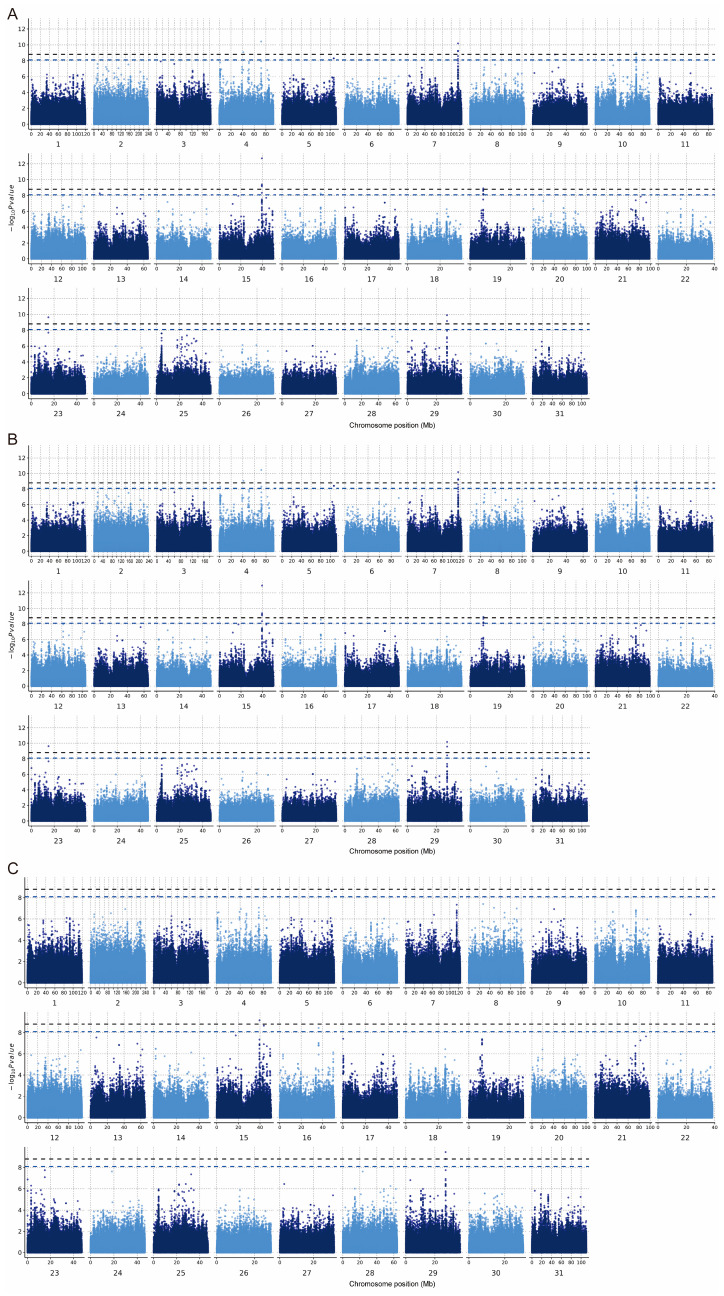
Manhattan plot of the CC trait. (**A**) GWAS conducted in PLINK. (**B**) GWAS conducted in GEMMA. (**C**) GWAS conducted in REGENIE. The black horizontal line indicates the threshold *p* < 1.61 × 10^−9^ and the blue line indicates *p* < 8.06 × 10^−9^.

**Figure 6:**
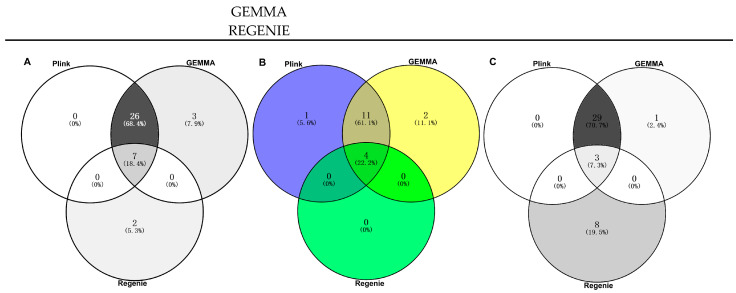
SNPs and genes significantly associated with the CC trait in different software: (**A**) correlated SNPs (*p* < 8.06 × 10^−9^); (**B**) correlated SNPs (*p* < 1.61 × 10^−9^); (**C**) correlated genes.

In the PLINK model, 33 significant SNP associations (*p* < 8.06 × 10^−9^) were found for the CC trait, of which 16 SNPs were highly significantly correlated with body size (*p* < 1.61 × 10^−9^). In GEMMA, 36 significant SNPs associations (*p* < 8.06 × 10^−9^) were found for the CC trait, of which 17 SNPs were significantly correlated with body size (*p* < 1.61 × 10^−9^). In REGENIE, nine significant SNPs associations (*p* < 8.06 × 10^−9^) were found for the CC trait, of which four SNPs were highly significantly correlated with body size (*p* < 1.61 × 10^−9^) (Figure 5, Table 2). In total, seven SNPs on chromosomes 4, 5, 15, 16, and 29 that were significantly (*p* < 8.06 × 10^−9^) associated with the CC trait appeared in the results of all three different analyses (Figure 6A). Additionally, four SNPs on ECA 4, 15, and 29 that were highly significantly (*p* < 1.61 × 10^−9^) associated with CC traits appeared in the results of all three different analyses (Figure 6B). Among the significant results, 38 SNPs were associated with the CC trait point in 41 interesting candidate genes (Figure 6C).

## 4. Discussion

Chinese donkeys are rich in genetic resources and can be divided into three types according to their body size: large, medium, and small [28]. The Xinjiang donkey is a small, dual-purpose local breed. It has important value in terms of meat, dairy, and skin production. However, the selection and breeding of Xinjiang donkeys are relatively slow, and marker-assisted breeding can greatly speed up the breeding process of the Xinjiang donkey. In total, 41 genes were found by means of GWAS using three different software packages.

We performed GWAS for the body size phenotype (CC) in the Xinjiang donkey population. The associations between SNPs and CC traits were analyzed using three previously reported methods: PLINK, GEMMA, and REGENIE [54,55,56]. A typical WGAS, currently including hundreds of thousands of SNPs, genotyped for thousands of individuals, represents a dataset that is several orders of magnitude larger than the datasets reported in previous linkage and association studies. As such, WGASs present new computational and statistical challenges. Perhaps the most apparent challenge is related to the increased burden of multiple testing: the concern that, from a set of hundreds of thousands of tests, many highly significant results are expected by chance alone, making it hard to distinguish signal from noise. Purcell et al. [50] developed a user-friendly software tool, PLINK, to facilitate the analysis of whole-genome data in several ways: by addressing the mundane but important need for easy ways to manage such data, by making routine analyses computationally efficient, and by offering new analyses that take advantage of whole-genome coverage. When considering a relatively small WGAS dataset of 100,000 SNPs genotyped for 350 individuals, for example, PLINK takes ~10 s to load, filter, and perform association analysis for all SNPs. PLINK fulfills two analytical needs: aiding the process of performing quality control (QC) on large datasets; and providing basic statistical tools to analyze variants in genetic models [57]. Linear mixed models have attracted considerable attention recently as a powerful and effective tool for accounting for population stratification and relatedness in genetic association tests. However, existing methods for the exact computation of standard test statistics are computationally impractical for even moderate-sized genome-wide association studies. To address this issue, Zhou et al. [52] presented an efficient and exact method, which they refer to as genome-wide efficient mixed-model association (GEMMA), which makes approximations unnecessary in many contexts. Mbatchou et al. [51] presented a novel machine-learning method called REGENIE for fitting a whole-genome regression model for quantitative and binary phenotypes that is substantially faster than the alternatives for performing multi-trait analyses, while maintaining statistical efficiency. This program was based primarily on saddle point approximation (SPA), The SPA method approximates the score test statistic by using the entire cumulant-generating function, rather than the first two moments (mean and variance) used with the normal approximation. REGENIE uses the polygenic effect estimates to control for population and family structure. This is why only nine SNPs were found to be significant using the REGENIE program, while 33 and 36 SNPs were found to be significant when using the PLINK and GEMMA programs, respectively. REGENIE produces rather conservative results. Recently, Gurinovich et al. [58] proposed the use of REGENIE to perform genome-wide association studies of binary traits in correlated data. In their paper, REGENIE appeared to produce slightly more conservative *p*-values in New England Centenarian Study-imputed genotype data, and substantially more conservative *p*-values in Long Life Family Study WGS data.

Five regions and eight SNPs were identified as having significant effects on the three methods in the GWAS (Figure 5). The *Equus asinus* (ass) autosome 7 (EAA 7) (115,561,781–115,604,466 bp) region, EAA10 (68,227,724–68,267,701), EAA15 (39,516,646–39,564,922), EAA19 (6,536,246–6,580,233), and EAA29 (27,390,749–27,421,411) were found to be significant with respect to CC traits, and are reported for the first time in this study. One SNP at 39,553,358 bp in nuclear factor of activated T cells 2 (*NFATC2*) on EAA 15 was identified to have a highly significant effect on CC using the three methods. According to Huang et al. [59], this gene is a direct regulatory transcription factor for osteoclast development that regulates osteoclast genesis and the expression of osteolysis-associated molecules, including matrix metalloproteinase (MMP)-9 and cathepsin K. Additionally, skeletal muscle formation and growth require the fusion of myoblasts to form multi-nucleated myofibers or myotubes [60]. Horsley et al. [61] demonstrated that nuclear addition and an increase in myotube size were controlled by a molecular pathway regulated by *NFATC2.* Calcium is a necessary factor for bone cell proliferation and differentiation. It has been shown that PGF2alpha receptor increase intracellular calcium levels, and the involvement of the calcium-regulated transcription factor nuclear factor of activated T cells (NFAT) in mediating PGF2alpha-enhanced cell growth was further investigated. The results showed that *NFAT* was activated by PGF2alpha and that *NFATC2* is required for PGF2alpha-induced muscle cell growth and nuclear accretion [62]. *NFATC2* is essential for regulating myonuclear cell addition and subsequent muscle growth. Valerie determined that *NFATC2* regulates the expression of IL-4, a novel molecular signal that controls myoblast fusion with myotubes, during muscle growth. IL-4 is expressed by a subset of muscle cells in fusing muscle cultures and acts through the IL-4 receptor on myoblasts to promote myoblast fusion and muscle growth [63]. The *NFATC2* gene is mainly involved in the WNT signaling pathway. Wnt signaling occurs through evolutionarily conserved pathways that affect cellular proliferation and fate decisions during development and tissue maintenance [64]. This fact may also be the underlying reason for the correlation between CC and body weight.

One SNP at 26419804 bp in PROP paired-like homeobox 1 (*PROP1*) on EAA 9 was an important SNP. The *PROP1* gene encodes a protein that regulates growth and development in mammals, and the *PROP1* gene is a novel important candidate gene for detecting genetic variation and growth, reproduction, metabolism traits selection and breeding [65]. Genetic variation in ovine *PROP1* was studied in 670 New Zealand Romney sheep; three single nucleotide polymorphisms (SNPs) were detected, and association analysis revealed that the variants were significantly associated with weaning weights [66]. Previous reports have demonstrated an association between PROP1, fertility, and growth traits in cattle [67]. In horses, this gene was inferred to be a signal of selection that could result in distinguishable phenotypes in Jeju horses and thoroughbred populations [68]. It is well known that a product of the *PROP1* gene regulates the expression of *PRL* and *POU1F1* genes. Previous studies have proved that *POU1F1* gene polymorphisms are significantly associated with the chest circumference of Nanyang cattle [69]. Similarly, *POU1F1* gene polymorphisms are associated with chest circumference in goats [70]. Therefore, this gene may be related to the CC of donkeys.

The association with CC on EAA 13 implicated a candidate gene, Ubiquitin B (*UBB*). It has been reported in a previous study that the loss of *UBB* can lead to a progressive degenerative disorder affecting neurons within the arcuate nucleus of the hypothalamus. This neurodegenerative cytopathology is accompanied by impaired hypothalamic control of energy balance and obesity [71].

One SNP (13871622) was located near the heart and the neural crest derivative 2 (*HAND2*) gene, which is a critical transcription factor for post-mitotic maintenance of the sympathetic nervous system [72]. Rodrigues et al. [73] found that selective expression of *HAND2* regulates skeletal muscle sympathetic and motor innervation, improving acetylcholine receptor (AChR) stability and nerve-activated muscle force generation. We found the *HAND2* gene to be enriched upon transcriptional regulation by the Runt-related transcription factor 2 (*RUNX2*) pathway. Previous studies have demonstrated a significant association between a 12 bp insertion within the *RUNX2* gene and chest circumference (*p* = 0.005) in Shaanbei white cashmere goats [74]. Here, we hypothesize that *HAND2* regulates chest circumference by directly promoting the transcription factor *RUNX2*. However, little is known about how *LOC123277442, LOC123282041, LOC106844757, LOC123278496*, and *LOC123278509* regulate the CC traits. The functions of these genes are unknown.

## 5. Conclusions

The GWAS performed in this study revealed the presence of genomic regions and putative causal genes associated with CC traits in the Xinjiang donkey. A total of 38 SNPs related to the CC of Xinjiang donkeys were identified. In addition, 41 candidate genes were identified, and several genes, including *NFATC2*, *PROP1*, *UBB*, and *HAND2*, may be the main candidate genes affecting the chest circumference of Xinjiang donkeys. More research is needed to verify the extent of the associations detected in this study and to determine the potential of using these genes to improve meat production performance in Xinjiang donkeys.

## Figures and Tables

**Table 1:** Statistics for the chest circumference trait of Xinjiang donkeys.

Parameter	Chest Circumference Trait
N (animals)	112
Mean (±SD)	151.50 (±12.80)
Minimum value (cm)	130.00
Maximum value (cm)	180.00
skewness	0.17
kurtosis	−0.70
Coefficient of variation (%)	8.44

**Table 2:** Single nucleotide polymorphism associations with the chest circumference trait (*p* < 1.61 × 10^−9^).

Chromosome	Model	Position (bp)	*p*-Value	Genes
4	PLINK	41,200,387	8.75 × 10^−10^	*LOC123285233* *LOC106834745*
	GEMMA			*LOC106834724* *DCAF17*
4	PLINK GEMMA REGENIE	71,906,099	3.73 × 10^−11^	/
7	PLINK GEMMA	115,561,781	5.88 × 10^−10^	/
7	PLINK GEMMA	115,566,944	6.73 × 10^−10^	/
7	PLINK GEMMA	115,604,466	6.44 × 10^−11^	
10	PLINK GEMMA	68,266,479	1.02 × 10^−9^	
10	PLINK	68,267,701	1.58 × 10^−9^	
15	PLINK GEMMA	39,516,646	5.58 × 10^−10^	*LOC123277442*
15	PLINK GEMMA	39,549,650	6.83 × 10^−10^	*NFATC2*
15	PLINK GEMMA REGENIE	39,553,358	2.08 × 10^−13^	*NFATC2*
15	PLINK GEMMA	39,564,922	4.03 × 10^−10^	*NFATC2*
19	PLINK GEMMA	6,580,233	1.28 × 10^−9^	*LOC106844757* *LOC123278496* *LOC123278509*
19	GEMMA	6,569,378	1.91 × 10^−9^	*LOC106844757* *LOC123278496* *LOC123278509*
19	GEMMA	6,575,744	1.91 × 10^−9^	*LOC106844757* *LOC123278496* *LOC123278509*
23	PLINK GEMMA	15,037,791	2.41 × 10^−10^	*LOC106824713* *LOC106824686* *LOC106824712* *LOC123280196*
24	PLINK GEMMA	18,270,706	1.32 × 10^−9^	/
29	PLINK GEMMA REGENIE	27,390,749	1.26 × 10^−10^	*LOC123282041*
29	PLINK GEMMA REGENIE	27,420,790	7.10 × 10^−10^	*LOC123282041*

## Data Availability

The raw data is in Supplementary Materials.

## Supplementary Materials

The following supporting information can be downloaded at: https://www.mdpi.com/article/10.3390/genes14051081/s1. Table S1: Statistics of sequencing data evaluation; Table S2: Statistics of coverage depth and coverage ratio; Table S3: Statistics of comparison result; Table S4: Relationship between SNPs and genes.
