# Supplementary material for: A Genome-Wide Association Study of the Chest Circumference Trait in Xinjiang Donkeys Based on Whole-Genome Sequencing Technology
Source: Genes (Basel). 2023 May 14;14(5):1081. doi: 10.3390/genes14051081 (PMC10217852; doi:10.3390/genes14051081)
Supplement: Supplementary file 1 [file genes-14-01081-s001.zip › Table S3.pdf]

Table S3. Statistics of comparison result.

Sample	Reads	Mapped (%)	Properly mapped (%)
89	171073244	170545049(99.69%)	164656522(96.25%)
90	188988536	188386500(99.68%)	181914952(96.26%)
91	182360468	181841412(99.72%)	176187066(96.61%)
92	178814508	178287424(99.71%)	172519696(96.48%)
93	168333730	166481801(98.90%)	160060282(95.09%)
94	173409684	172870888(99.69%)	167085060(96.35%)
95	172290474	171778658(99.70%)	166059870(96.38%)
96	171728818	171199614(99.69%)	165719862(96.50%)
97	181479318	180850008(99.65%)	174847004(96.35%)
98	168330842	167857761(99.72%)	162303392(96.42%)
99	175156466	174621967(99.69%)	168714736(96.32%)
100	173686952	173123087(99.68%)	166940302(96.12%)
101	170680666	170164650(99.70%)	164830382(96.57%)
102	186405440	185780861(99.66%)	178292720(95.65%)
103	175163936	174628746(99.69%)	168507700(96.20%)
104	175964222	175447210(99.71%)	169427278(96.29%)
105	179959592	179419397(99.70%)	173748174(96.55%)
106	171609630	171057104(99.68%)	165578586(96.49%)
107	178926654	178372701(99.69%)	172947370(96.66%)
108	175799964	175264938(99.70%)	169676688(96.52%)
109	168679510	168156583(99.69%)	162328646(96.23%)
110	174959176	174412500(99.69%)	168516914(96.32%)
111	185549896	184956369(99.68%)	178742998(96.33%)
112	178582460	178032292(99.69%)	172108766(96.37%)
1	170554048	170040678(99.70%)	163900128(96.10%)
2	182393944	181830056(99.69%)	175628304(96.29%)
3	175640224	175145227(99.72%)	168980872(96.21%)
4	194353124	193785352(99.71%)	186206934(95.81%)
5	188132368	187589416(99.71%)	179968718(95.66%)
6	177115530	176580700(99.70%)	170184018(96.09%)
7	177822294	177328195(99.72%)	170266420(95.75%)
8	174698058	174153086(99.69%)	167992488(96.16%)
9	174770296	174212577(99.68%)	168050292(96.15%)
10	189986666	189442359(99.71%)	182175562(95.89%)
11	175245862	174696617(99.69%)	167745022(95.72%)
12	166363718	165833750(99.68%)	160202986(96.30%)
13	175657480	175136868(99.70%)	168303162(95.81%)
14	176357478	175834674(99.70%)	170113122(96.46%)
15	174361602	173799856(99.68%)	167430464(96.02%)
16	173686486	173140692(99.69%)	167122946(96.22%)
17	172797294	172238937(99.68%)	165855304(95.98%)
18	184888924	184343519(99.71%)	177634722(96.08%)

19	174711354	174141799(99.67%)	167769836(96.03%)
20	167037622	166530282(99.70%)	160445458(96.05%)
21	168649776	168115149(99.68%)	162565416(96.39%)
22	175509328	174952799(99.68%)	168809476(96.18%)
23	181087136	180537602(99.70%)	174184126(96.19%)
24	169282012	168740648(99.68%)	162556404(96.03%)
25	176909374	176338073(99.68%)	170037010(96.12%)
26	172437308	171869561(99.67%)	166097538(96.32%)
27	201268320	200633579(99.68%)	193638012(96.21%)
28	182579078	182039315(99.70%)	175481648(96.11%)
29	232900960	232189122(99.69%)	224104630(96.22%)
30	171966122	171434148(99.69%)	165958704(96.51%)
31	173607070	173046923(99.68%)	166532454(95.92%)
32	171903232	171389571(99.70%)	165579686(96.32%)
33	174809518	174259577(99.69%)	168620940(96.46%)
34	168193974	167688333(99.70%)	162326934(96.51%)
35	166348390	165764724(99.65%)	160099106(96.24%)
36	176327558	175762100(99.68%)	169970950(96.40%)
37	196667522	196103674(99.71%)	190047406(96.63%)
38	198367484	197814308(99.72%)	191656620(96.62%)
39	192242592	191630016(99.68%)	185872962(96.69%)
40	171496190	170963689(99.69%)	165751812(96.65%)
41	174589360	174062209(99.70%)	167868198(96.15%)
42	180354284	179840589(99.72%)	174627708(96.82%)
43	178380732	177787788(99.67%)	171518002(96.15%)
44	170881896	170349918(99.69%)	164999034(96.56%)
45	174496234	173935286(99.68%)	168664180(96.66%)
46	169257200	168766876(99.71%)	163522588(96.61%)
47	187009736	186430993(99.69%)	180620442(96.58%)
48	183347100	182774985(99.69%)	177181352(96.64%)
49	177440496	176903457(99.70%)	171006944(96.37%)
50	180447062	179856663(99.67%)	173731522(96.28%)
51	171587872	171075039(99.70%)	164448496(95.84%)
52	172988810	172468811(99.70%)	166341808(96.16%)
53	171571768	171038102(99.69%)	164973828(96.15%)
54	177470026	176951208(99.71%)	171436560(96.60%)
55	171346160	170845566(99.71%)	164946546(96.27%)
56	175812276	175291592(99.70%)	169596316(96.46%)
57	169812328	169284264(99.69%)	163136912(96.07%)
58	165939584	165443467(99.70%)	160059064(96.46%)
59	174175700	173659329(99.70%)	168511736(96.75%)
60	171146614	170664322(99.72%)	165489964(96.69%)
61	187423684	186856247(99.70%)	180421290(96.26%)
62	195398398	194821528(99.70%)	186816594(95.61%)

63	173431852	172887723(99.69%)	167184242(96.40%)
64	182036932	181505709(99.71%)	173018596(95.05%)
65	180276310	179727264(99.70%)	172777448(95.84%)
66	170961392	170423091(99.69%)	164492694(96.22%)
67	169305134	168735682(99.66%)	163290362(96.45%)
68	181058306	180484438(99.68%)	174161072(96.19%)
69	170248958	169751854(99.71%)	163868058(96.25%)
70	172595124	172076151(99.70%)	166519568(96.48%)
71	197179840	196567118(99.69%)	189516280(96.11%)
72	168018820	167543563(99.72%)	162718214(96.85%)
73	188071110	187507833(99.70%)	180737970(96.10%)
74	191642878	191078838(99.71%)	184199560(96.12%)
75	182369882	181849202(99.71%)	175600522(96.29%)
76	172095080	171587371(99.70%)	165758960(96.32%)
77	170550126	170053460(99.71%)	163453706(95.84%)
78	199932648	199351041(99.71%)	192572378(96.32%)
79	170580926	170087557(99.71%)	164734702(96.57%)
80	170021000	169520937(99.71%)	163880230(96.39%)
81	173611754	173106488(99.71%)	166904950(96.14%)
82	170244522	169751041(99.71%)	164374114(96.55%)
83	197858378	197252556(99.69%)	190634894(96.35%)
84	170327920	169790170(99.68%)	164481728(96.57%)
85	179834876	179322344(99.71%)	172597336(95.98%)
86	167043808	166569706(99.72%)	160749186(96.23%)
87	177202394	176698827(99.72%)	170338412(96.13%)
88	183666244	183120510(99.70%)	176613860(96.16%)
