# Supplementary material for: A Genome-Wide Association Study of the Chest Circumference Trait in Xinjiang Donkeys Based on Whole-Genome Sequencing Technology
Source: Genes (Basel). 2023 May 14;14(5):1081. doi: 10.3390/genes14051081 (PMC10217852; doi:10.3390/genes14051081)
Supplement: Supplementary file 1 [file genes-14-01081-s001.zip › Table S4.pdf]

Table S4. Relationship between SNPs and genes.

Type	SNP number
Intergenic	5,201,198
Intron	4,738,320
Upstream	898,713
Downstream	730,993
UTR_3_prime	118,007
Intragenic	91,113
CDS synonymous coding	79,991
CDS non synonymous coding	74013
Exon	53,095
UTR_5_prime	42,707
Start gained	8,878
CDS Stop gained	1,128
Splice site donor	684
Splice site acceptor	584
Start lost	204
Stop lost	172
Synonymous stop	69
Non synonymous start	40
